# The Surgical Treatment for Spinal Intradural Extramedullary Tumors

**DOI:** 10.4055/cios.2009.1.3.165

**Published:** 2009-08-17

**Authors:** Dong-Ki Ahn, Hoon-Seok Park, Dae-Jung Choi, Kwan-Soo Kim, Tae-Woo Kim, Soon-Youl Park

**Affiliations:** Department of Orthopedic Surgery, Seoul Sacred Heart General Hospital, Seoul, Korea.

**Keywords:** Intradural extramedullary tumor, Surgical treatment, Prognosis

## Abstract

**Background:**

We wanted to investigate the results of surgical treatment and analyze the factors that have an influence on the neurologic symptoms and prognosis of spinal intradural extramedullary (IDEM) tumors.

**Methods:**

The spinal IDEM tumor patients (11 cases) who had been treated by surgical excision and who were followed up more than 1 year were retrospectively analyzed. Pain was evaluated by the visual analogue scale (VAS) and the neurologic function was assessed by Nurick's grade. The pathological diagnosis, the preoperative symptom duration, the tumor location on the sagittal and axial planes and the percentage of tumor occupying the intradural space were investigated. In addition, all these factors were analyzed in relation to the degree of the preoperative symptoms and the prognosis. On the last follow-up, the MRI was checked to evaluate whether or not the tumor had recurred.

**Results:**

The most common diagnosis was schwannomas (73%), followed by meningiomas (18%). The percentage of tumor occupying the intradural space was 82.9 ± 9.4%. The VAS score was reduced in all cases from 8.0 ± 1.2 to 1.2 ± 0.8 (*p* = 0.003) and the Nurick's grade was improved in all cases from 3.0 ± 1.3 to 1.0 ± 0.0 (*p* = 0.005). The preoperative symptoms were correlated with only the percentage of tumor occupying the intradural space (VAS; r^2^ = 0.75, *p* = 0.010, Nurick's grade; r^2^ = 0.69, *p* = 0.019). One case of schwannoma recurred.

**Conclusions:**

The degree of neurologic symptoms was correlated with the percentage of tumor occupying the intradural space. All the tumors were able to be excised through the posterior approach. The postoperative neurologic recovery was excellent in all the cases regardless of any condition. Therefore, aggressive surgical excision is recommended even for cases with a long duration of symptoms or a severe neurologic deficit.

Intradural extramedullary (IDEM) tumors account for two thirds of all primary intraspinal neoplasms and these include schwannomas, meningiomas and ependymomas.1) While the clinical symptoms and pathology of IDEM tumors have not changed, there have been dramatic improvements in the diagnosis and treatment with the advances of the radiological and surgical techniques. Particularly, myelography, CT and MRI allow making an easy diagnosis and accurate identification of the location of a tumor in the dura mater and its dural attachment before surgery, and a significantly improved surgical outcome can be expected with the use of an operating microscope.^1^-3)

However, given the high sensitivity of the radiological techniques such as MRI, care should be taken not to overlook a tumor in the upper spine when observing radiographic evidence of nerve root compression in the lower lumbar region.

In this study, we assessed the IDEM tumors that we surgically managed with regard to the pathological diagnosis, the preoperative medical history and the clinical symptoms, the surgical treatment outcome and recurrence, the prognostic factors and the reasons for a misdiagnosis.

## METHODS

**Fig. 1:**
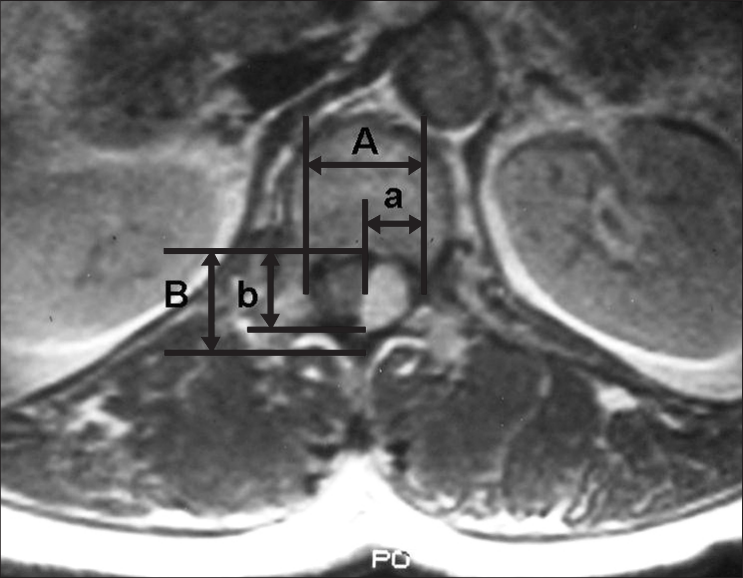
This picture shows how to calculate the percentage of tumor occupying the intradural space on an axial MRI film. It is as follows: {(a + b) / (A + B)} × 100. A: transverse diameter of the intradural space, B: longitudinal diameter of the intradural space, a: transverse diameter of the tumor mass, b: longitudinal diameter of the tumor mass.

Among the patients who had been surgically treated for IDEM tumors by the first author of this study between May 2002 and March 2007, those patients who were available for MRI reexaminations after the at least 1 year follow-up period were included in this retrospective study. Each patient's medical history, radiological examination record and the findings of the physical examination that were performed in an outpatient setting were investigated. There were 10 patients (11 cases; male:female = 3 : 7) and their mean age was 57 years (range, 32 to 72 years). One patient had two tumors. The preoperative duration of symptoms and the symptomatic characteristics were examined. Preoperative lower limb pain was assessed by using a 10-point visual analogue scale (VAS). For the assess ment of the preoperative neurological function, the patients' ambulation ability was graded into 5 levels according to the Nurick's grading system (Table 1).4) For the evaluation of postoperative recovery, the patients' neurological symptoms were observed during an inquiry and the physical examinations were graded. The location of a tumor on the sagittal and axial MRI images and the percentage of the tumor that occupied the intradural space were investigated to find any associations and to determine the statistical significance with the preoperative symptoms and the final surgical outcome. The percentage of tumor occupying the intradural space was calculated on the axial image showing the maximum size as follows (Fig. 1): {(the transverse diameter of the tumor mass + the longitudinal diameter of the tumor mass) / (the transverse diameter of the intradural space + the longitudinal diameter of the intradural space)} × 100. The diagnosis of recurrence was based on the MRI scans that were performed at the last follow-up. The SPSS ver. 11.5 (SPSS Inc., Chicago, IL, USA) was used for statistical analysis and the Wilcoxon signed rank test, the partial correlation test, the Spearman correlation test, the Mann Whitney test and ANOVA were also performed.

### Operative Technique and Postoperative Treatment

**Fig. 2:**
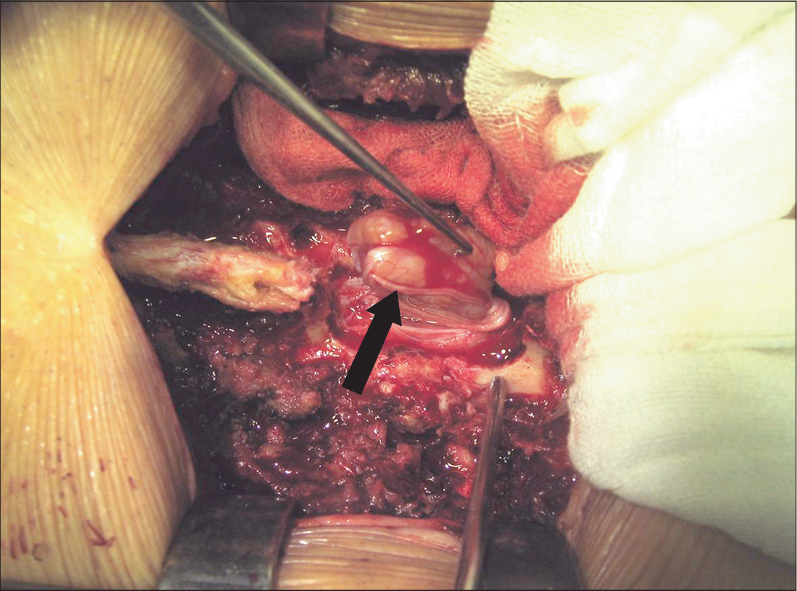
This shows a nerve fiber over the surface of schwannoma. It was separable from the tumor mass.

**Fig. 3:**
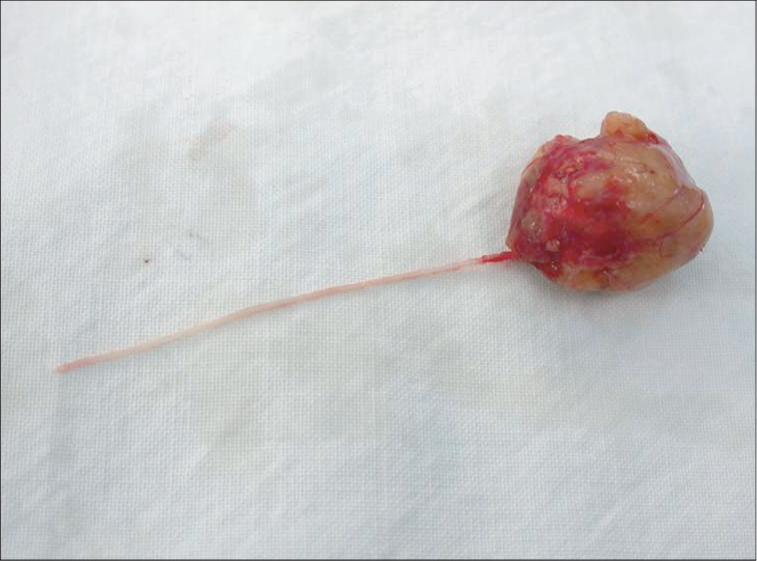
This shows a nerve fiber that penetrated a schwannoma. But any distal connected fiber was not identified.

Laminectomy and tumor resection were performed through a posterior approach in all cases, and this was irrespective of the location of a tumor. Posterior spinal fusion was additionally carried out to prevent postoperative instability for the 5 cases of tumors which was located at the distal part of the thoracolumbar junction. Posterior spinal fusion was not applied in a 32-year-old male with a schwannoma at the level of L3 because the bilateral facet joints could be preserved due to the tumor's location (posterior to the cauda equina), no degenerative changes were noted in the anterior intervertebral discs and the posterior muscles were not weak. During the removal of a cervical tumor ventral to the spinal cord, the paired denticulate ligaments on one side were incised to allow lateral retraction of the spinal cord. The spinal cord or the cauda equina was protected with a cottonoid and the tumor was removed with a freer and a hook. During the excision, the nerve fibers over the surface of a tumor were carefully separated (Fig. 2) and those penetrating the tumor mass were either excised with the tumor if the distal ends were not identified (Fig. 3) or they were preserved through an intralesional tumor excision if the distal ends were observable. A total of 7 (64%) marginal and 4 (36%) intralesional excisions were performed (Table 2). Closure of the dura mater was performed with prolene 7-0 in the first 3 cases, but prolene 6-0 reinforced with fibrin glue was used in the rest of the cases. Negative pressure drainage was performed in all the cases. The drain was removed on the 2nd postoperative day and gait training was started on the 3rd postoperative day. A thoracolumbosacral orthosis was worn for 2 postoperative months by the patients who underwent posterior spinal fusion with instrumentation.

## RESULTS

### Pathological Diagnosis

**Fig. 4:**
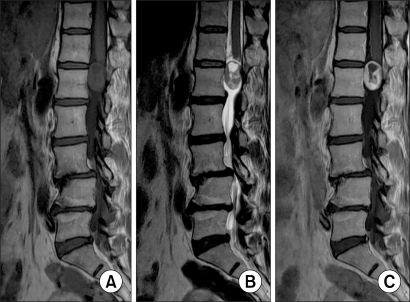
Schawannoma: (A) The T1 weighted image shows signal intensity that is similar to that of the spinal cord. (B) The T2 weighted image shows ir -regular and higher signal in tensity than that of the CSF. (C) The contrast-enhanced MR often shows an irregular margin and ring-shape enhancement.

**Fig. 5:**
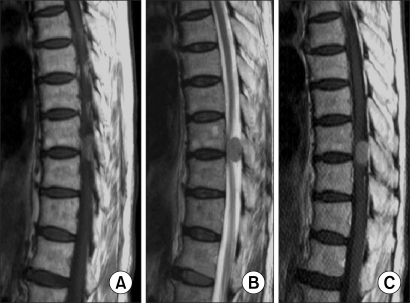
Meningioma: (A, B) The T1 & T2 weighted images both show slightly lower intense than that of the cord and this is a homogenous lesion. (C) Contrast-enhanced MR shows high homogenous signal intensity of tumor.

**Fig. 6:**
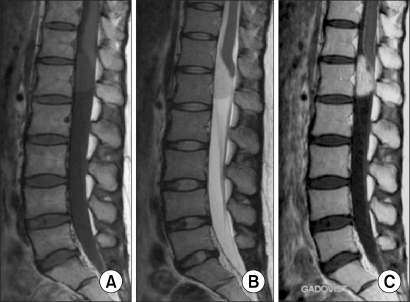
Ependymoma: (A) The T1 weighted image shows signal intensity that is similar to the spinal cord. (B) The T2 weighted image shows higher signal intensity than that of the spinal cord. (C) Contrast-enhanced MR shows a well defined margin and regular intensity lesion.

The pathological diagnoses included 8 cases of schwannoma (73%), 2 cases of meningioma (18%) and 1 case of ependymoma (9%). There was 1 case of multiple schwannomas and 9 single tumors (Table 2) (Figs. 4 to 6).

### Tumor Location and the Percentage of Tumor Occupying the Intradural Space

As observed on the sagittal plane images, 1 tumor was located in the cervical spine (9%), 4 in the thoracic spine (36%), 2 in the thoracolumbar spine (18%) and 4 in the lumbar spine (36%). With regard to the relative location, 6 were dorsal and 5 were ventral to the spinal cord or the cauda equina. The percentage of tumor occupying the intradural space was 82.9 ± 9.4% (Table 2).

### Symptoms

The mean duration of symptoms was 19.3 months (range, 1 to 120 months) and the mean postoperative follow-up period was 35.2 months (range, 12 to 72 months) (Table 2). The most common symptoms were lower limb pain and numbness, which were observed in all the cases. The symptoms tended to increase during walking rather than during bed rest and sitting. Motor weakness was also observed in 7 patients (70%).

All the cases' symptoms improved postoperatively. The VAS score decreased in all the cases from an average of 8.0 ± 1.2 (range, 7 to 10) preoperatively to an average of 1.2 ± 0.8 (range, 0 to 2) at the last follow-up (*p* = 0.003). The average Nurick's grade improved from 3.0 ± 1.3 (range, 1 to 5) preoperatively to 1.0 ± 0.0 (1.0) at the last follow-up (*p* = 0.005) (Table 3).

The preoperative symptoms measured by the VAS and the Nurick's grading system were highly associated with the percentage of the tumor occupying the intradural space. However, no statistically significant association or difference was found between the preoperative symptoms and the duration of symptoms and the location of a tumor (Table 4). A remarkable improvement in symptoms was obtained in all the cases after surgery.

### Complications

Duraplasty using an iliotibial band was performed in one case with a pseudo-meningocele as a complication at the 5th postoperative month. The internal fixation was removed in one case due to the patient's complaint of discomfort at the 14th postoperative month. Signs of radiological instability were noted proximal to the instrumented segments in one case, but this potential instability was not symptomatic. There was no deterioration of the neurological symptoms after surgery. Signs of instability were not observed in the cases of laminectomy without spinal fusion.

### Symptoms in Common with the Distal Lumbar Disorders

At the time of admission, the painful limping found in 2 patients was interpreted as a sign of other lower lumbar disorders based on the MRI scans: L5-S1 foraminal stenosis in one case and L4-L5 and L5-S1 isthmic spondylolisthesis in the other. Surgical treatments for these patients resulted in no symptomatic improvement and deterioration occurred instead. Accordingly, a new MRI scan was performed on the proximal segments and a tumor was found at the level of T9 in one case and at the level of T11 in the other.

### Recurrence

The MRI scan at the last follow-up showed the recurrence of schwannoma in one patient (9%).

## DISCUSSION

Previous reports have stated that about 5 females and 3 males out of 1,000,000 people are affected by primary spinal tumors every year and only 2/3 of them are IDEM tumors.5) Due to the rarity of IDEM tumors, it is not easy to enroll a large enough study population to assess a surgical procedure for treating such tumors. Accordingly, there have been large time gaps among the reports and studies that involve cases collected for more than a 10 year period or the reports have been focused on an analysis of the existing literature. In other words, it is difficult to find studies that are focused on a certain radiological examination technique or a surgical procedure.

As a historical note, Sir Victor Horsley in 1888 succeeded for the first time in surgically excising an IDEM tumor located in the thoracic region, and this was 44 years before the invention of myelography.2) Thereafter, the advancement in radiological examination techniques and the use of surgical microscopes has brought about remarkable improvements in the diagnosis and surgical treatment, but the basic surgical principles have not changed.3) Meningiomas account for 25-46% of all primary intraspinal neoplasms and spinal meningiomas are only 7.5-12.5% of all meningiomas because most meningiomas are found in the brain.6) Spinal meningiomas are mostly located in the thoracic vertebra and they are more common in females, which is presumably due to the influence of female hormones.6) In this study, two meningiomas were also found in the thoracic vertebra of female patients. With regard to the treatment of the dural attachment of a meningioma, there are three common procedures: 1) some portion of the dura mater is resected with the tumor to remove any residual tumor cells and then duraplasty is performed, 2) some of the internal dura mater is peeled off and the rest of it is sutured, or 3) the dural attachment is cauterized.7) In this study, the closures were performed without additional procedures because all the tumors were easily separated from the dura mater and no recurrence was observed at the last follow-up. With regard to the patient with multiple schwannomas, the 6-year preoperative medical history showed that the Nurick's grade changed from 1 to 2 over the period and the tumor size on the sagittal MRI images increased 2.38% per year at the level of T12 and 12.96% per year at the level of L3, which is contrary to the previous studies that described these schwannomas as benign, slow-growing tumors.8) Schwannomas have been known to grow with displacing nerve fibers laterally rather than invading them. Even so, complete removal of a schwannoma is only possible when the nerve fibers are also excised in the case of their involvement within the tumor. Most authors have emphasized that the preservation of nerve roots compromises achieving complete tumor removal.^9^-11) Yet according to Kim et al.12) only 23% of the complete excisions of schwannomas with functionally important nerve roots resulted in the development of neurological symptoms (not severe ones) because the nerve roots involved in the tumors had already become dysfunctional. In this study, nerve fibers were found traveling distally through the tumor mass in 1 out of 8 schwannomas and so intralesional excision was performed in this case. The nerve fibers with no identified distal ends were removed with the tumor mass by marginal resection in 2 cases. No deterioration of the neurological function was observed on the neurological examination that was performed immediately after surgery. Because the Nurick's grade improved to 1 in all the cases at the last follow-up, the nerve fibers with no identified distal end were deemed dysfunctional. Based on the observation of only 1 tumor recurrence on MRI, we believed that the preservation of nerve roots during tumor resection did not affect the recurrence of tumor.

In this study, a posterior approach was used in all the cases regardless of the location of a tumor in and relative to the spinal cord. According to the literature, 31% of the tumors are located ventral to the spinal cord13) and Slin'ko and Al-Qashqish14) claimed that an extreme lateral or an anterior approach was necessary for the removal of these tumors. However, extreme lateral approaches require spinal fusion due to the removal of the lamina and the facet joint, and the anterior approaches are difficult to use due to the epidural venous bleeding, the limited field of view and the removal of several vertebral bodies. In this current study, a posterior approach was used to preserve the bilateral facet joints and accordingly spinal fusion could be avoided, with excluding the lumbar and thoracolumbar tumors. We also experienced no difficulties in removing cervical and thoracic tumors ventral to the spinal cord and so we thought extreme lateral or anterior approaches were not necessary. For the removal of a cervical tumor, we cut the paired denticulate ligaments superior and inferior to the tumor to promote mobility of the spinal cord. However, we thought that it was safer to use an anterior approach when the tumor extended anteriorly to the surface of the dura mater. In 4 out of 11 cases, marginal excision was not performed for different reasons: we tried to preserve the nerve root involved in the tumor in 1 case of schwannoma, the tumor capsule was destroyed during release due to the limited accessibility to the tumor located ventral to the spinal cord in 1 case of schwannoma and 1 case of meningioma, and the tumor was not solid enough for marginal excision in case of ependymomas.15)

According to the previous reports, most of the neurological symptoms improved postoperatively in most of the cases, but partial improvements, no improvement and deterioration were also observed in some cases. With regard to the factors that influence the prognosis, the longer the preoperative duration of symptoms was, the severer the neurological deficits were,1) the more proximal the location of a tumor was2) and the more ventral the tumor was to the spinal cord, the worse the surgical outcome was.14) In this study, the degree of preoperative neurological symptoms was not associated with the duration of symptoms, the location of a tumor or the relative location of a tumor to the spinal cord. However, the preoperative neurological symptoms measured by the VAS and the Nurick's grading system were significantly related with the percentage of tumor occupying the intradural space. However, considering that the tumors of Nurick grade 4 and over were all observed in the thoracic region, this above mentioned correlation may be a consequence of statistical error due to the small patient population of our study. In addition, we thought that surgical intervention should be recommended for all IDEM tumors regardless of the prognostic factors because the Nurick's grade improved to 1 postoperatively for all the cases in our study regardless of the prognostic factors. Presumably, the advanced neurological deficits could recover because of the slower progress of nerve compression, as compared with other conditions that present with similar levels of neurological deficits, and provided that the nerve tissues have time enough to adjust.

According to el-Mahdy et al.2) the postoperative recurrence rate of IDEM tumors was 16%. According to Asazuma et al.9) the recurrence rate of intraspinal neoplasms was 7.2% and 46% of recurrenced masses were IDEM spinal tumors which recur more commonly than other intraspinal tumors. They also reported that the ventral location of a tumor, extradural invasion, neurogenic tumors and ependymomas were the risk factors for recurrence. According to the study on the treatment of ependymomas by Klekamp and Samii,16) the recurrence rate was 29.5% at 5 years after complete resection, and this rate was higher than that of other tumors. We believed incomplete removal of the dura mater, which is the origin of the tumor, caused the high rate of recurrence of meningioma. In this study, the recurrence rate was found to be 9.1%. The MRI at the last follow-up showed that the thoracic schwannoma that was intralesionally excised for its ventral location to the spinal cord relapsed despite the improvement of the neurological symptoms. However, it is our understanding that our obtained recurrence rate is not reliable because the mean follow-up period (37 months) was too short and various pathological diagnoses were included.

No neurological deficit occurred as a postoperative complication, but lower limb pain and headache appeared to increase in the patient with multiple schwannomas under such stimuli as walking, coughing and direct hitting on the surgical site from the 1st postoperative month. An MRI scan of the patient showed a 16 × 5 cm pseudomeningocele. During the reoperation, we thought that the untying and breakage of the prolene 7-0 used for the closure of the dura mater was the cause of the complication and so we used prolene 6-0 instead and fibrin glue for reinforcement.

Before the introduction of MRI, spinal cord tumors were often misdiagnosed as multiple sclerosis, syringomyelia or a herniated nucleus pulposus.17) The recent development of radiological examinations has helped to prevent such misdiagnoses, but thoracic tumors were overlooked and lumbar lesions were surgically treated in 2 cases of this study. According to Shin et al.18) as the clinical symptoms of a tumor are similar to those of the herniation of an intervertebral disc and spinal stenosis, taking a detailed history, conducting a thorough physical examination and performing MRI scans on the proximal regions are recommended for patients with back pain and radiating pain in the lower limbs. In this study, the surgical intervention for lesions that could result in neurological symptoms in the distal area did not lead to improvements of the symptoms. This was because we made a wrong diagnosis, but we didn't expect to find another lesion in the proximal area because a lesion in the distal area was definitely found in MRI scan and the physical examination did not reveal the compression of the central nervous system. The neurological symptoms were exacerbated following primary surgery in both of the patients. Recovery occurred after the removal of the proximal tumors. Therefore, we thought that the primary cause of the symptoms was not the distal nerve compression and so we made it a rule to take sagittal MRI images for the whole spinal column. However, we are not able to determine why the progress of the neurological symptoms accelerated following the primary surgery and we couldn't find any literature to refer to.

The most prevalent spinal IDEM tumor was schwannoma. The degree of preoperative symptoms was associated with the percentage of tumor occupying the intradural space. Postoperatively, remarkable improvements in neurological deficits were achieved regardless of the percentage of tumor occupying the intradural space, the degree of preoperative symptoms and the duration of symptoms. Therefore, surgical intervention is also recommended for spinal IDEM tumors with prolonged or severe neurological symptoms.

For making the diagnosis of IDEM tumors, taking a detailed history, a through physical examination and performing MRI scans on the proximal regions are recommended because the symptoms of IDEM tumors can be similar with those of lumbar herniated nucleus pulposus and spinal stenosis.

## Figures and Tables

**Table 1:**
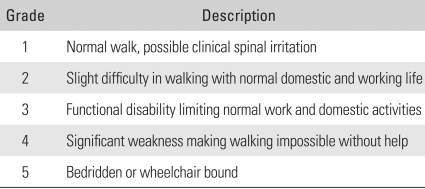
Nurick's Functional Grading

**Table 2:**
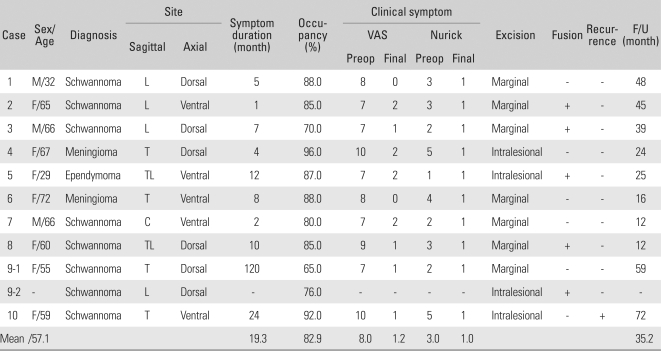
The Demographic, Clinical and Radiological Data

VAS: Visual analogue scale, L: Lumbar, T: Thoracic, TL: Thoracolumbar, C: Cervical, F/U: Follow up.

**Table 3:**
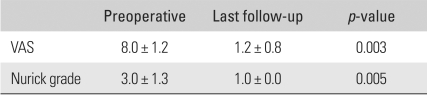
Clinical Improvement

VAS: Visual analogue scale.

**Table 4:**
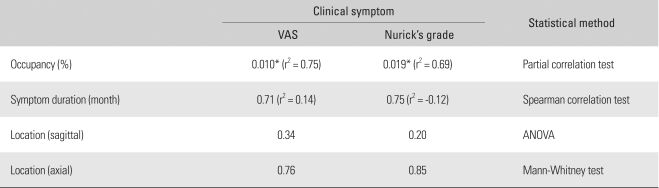
Influencing Factors to Clinical Symptoms

^*^Significant, VAS: Visual analogue scale.
